# RETRACTION: KIF23 Promotes Gastric Cancer by Stimulating Cell Proliferation

**DOI:** 10.1155/dim/9856819

**Published:** 2025-07-21

**Authors:** Disease Markers

RETRACTION: X.-L. Li, Y.-M. Ji, R. Song, X.-N. Li, and L.-S. Guo. “KIF23 Promotes Gastric Cancer by Stimulating Cell Proliferation,” *Disease Markers* 2019, no. 1 (2019): 1–9, https://doi.org/10.1155/2019/9751923.

The above article, published online on 17 March 2019 in Wiley Online Library (wileyonlinelibrary.com), has been retracted by John Wiley & Sons Ltd.

The retraction has been agreed following an investigation of the concerns raised by *Hoya camphorifolia* on PubPeer [1], which identified multiple instances of inappropriately overlapping figures.

Specifically:

- Figure 3c: The Western blot panels for Ki67 and β-actin are identical to the bands corresponding to RAB38 and β-actin in Figure 2b of [2].
- Figure 4a: Some of the tumors appear in Figure 5a of [3] and Figure 4a of [4].
- Figure 4c,d: Multiple duplication of the immunohistochemistry images, spanning at least 3 other publications by different author groups [5–7].

As a result of the investigation, the data and conclusions of this article are considered unreliable.

Lan-Shuan Guo disagrees with this retraction. The other authors were informed of this decision but did not provide a response.
